# Medical care costs incurred by patients with smoking-related non-small cell lung cancer treated at the National Cancer Institute of Mexico

**DOI:** 10.1186/s12971-014-0025-4

**Published:** 2015-02-04

**Authors:** Oscar Arrieta, Roger Humberto Quintana-Carrillo, Gabriel Ahumada-Curiel, Jose Francisco Corona-Cruz, Elma Correa-Acevedo, Juan Zinser-Sierra, Dolores de la Mata-Moya, Alejandro Mohar-Betancourt, Vicente Morales-Oyarvide, Luz Myriam Reynales-Shigematsu

**Affiliations:** Clinic of Thoracic Oncology, National Cancer Institute of Mexico (INCan), Mexico City, Mexico; Tobacco Control Research Department, Center for Population Health Research, National Institute of Public Health, Cuernavaca, Mexico; Medical Oncology Department, National Cancer Institute of Mexico (INCan), Mexico City, Mexico; Head of the Epidemiology Unit, National Cancer Institute of Mexico (INCan), Mexico City, Mexico; National Autonomous University of Mexico (UNAM), Mexico City, Mexico

**Keywords:** Non-small cell lung cancer (NSCLC), Tobacco, Medical care cost, Chemotherapy cost

## Abstract

**Background:**

Smoking is a public health problem in Mexico and worldwide; its economic impact on developing countries has not been well documented. The aim of this study was to assess the direct medical costs attributable to smoking incurred by lung cancer patients treated at the National Cancer Institute of Mexico (INCan).

**Methods:**

The study was conducted at INCan in 2009. We carried out a cost of illness (COI) methodology, using data derived from an expert panel consensus and from medical chart review. A panel of experts developed a diagnostic-therapeutic guide that combined the hospital patient pathways and the infrastructure, human resources, technology, and services provided by the medical units at INCan. Cost estimates in Mexican pesos were adjusted by inflation and converted into US Dollars using the 2013 FIX exchange rate for foreign transactions (1 USD = 13.06 Mexican pesos).

**Results:**

A 297 incident cases diagnosed with any type of lung cancer were analyzed. According to clinical stage, the costs per patient were 13,456; 35,648; 106,186; and 144,555 USD, for lung cancer stages I, II, III, and IV respectively. The weighted average annual cost/patient was and 139,801 USD and the average annual cost/patient that was attributable to smoking was 92,269 USD. This cost was independent of the clinical stage, with stage IV representing 96% of the annual cost. The total annual cost of smoking-related lung cancer at INCan was 19,969,781 USD.

**Conclusions:**

The medical care costs of lung cancer attributable to smoking represent a high cost both for INCan and the Mexican health sector. These costs could be reduced if all provisions established in the Framework Convention of Tobacco Control of the World Health Organization were implemented in Mexico.

## Introduction

Smoking is a worldwide primary public health problem. It has been estimated that approximately 1.2 billion people smoke worldwide, and half of them will die of diseases caused by smoking [1]. Currently, close to six million smoking-related deaths occur per year. It has been calculated that this number will increase to eight million deaths by the year 2030, of which 80% will occur in low- and medium-income countries [2].

The harmful effects of smoking on individual and population health, as well as its economic consequences, are well described in the international bibliography [3-7].

However, its effects on the economy of developing countries have not been properly documented, creating a shortage of scientific information for promoting the correct implementation of the Framework Convention of Tobacco Control of the World Health Organization (WHO-FCTC) in Mexico [8].

Lung cancer (LC) is the main cancer-related cause of death worldwide in both men and women [9-11]. It has been estimated that in 2013, there will be 228,190 new LC cases (118,080 in men and 110,110 in women) and a total of 159,480 deaths due to LC (87,260 in men and 72,220 in women) in the United States [9]. Considering that up to 85% of these tumors are associated with smoking, their incidence has increased in a sustained fashion since 1970. This increase is attributed to the increase in smoking, especially among women [10,12-16]. A total of 9,148 new cases and 8,807 deaths due to LC were registered in Mexico in 2008 [10].

LC is classified into two large groups: small cell (SCLC) and non-small cell (NSCLC) lung cancer. The latter represents close to 90% of total LC cases, of which more than 95% of cases are diagnosed at advanced stages in our country [17,18] and have only a 16% 5-year survival rate [10,15,17-20]. While the research and development of new therapeutic schemes improve patient quality of life and survival, they also cause a dramatic increase in treatment costs [21-24].

The National Cancer Institute of Mexico (INCan, for its initials in Spanish) works as the reference site for patients with the most important types of cancer at a national scale. It is implemented with all the necessary human and material resources to provide comprehensive and high quality care for cancer patients. The goal of this study was to assess the direct medical care costs of NSCLC at the INCan that are attributable to smoking.

## Materials and methods

### Study population

To estimate the direct health care costs of Lung cancer at Incan*, we carried out a cost of illness (COI) methodology, using incidence approach. During 2009, 297 incident cases with any type of NSCLC were admitted. The patient inclusion criteria were as follows: ≥35 years of age, current/past smokers and non-smokers at the time of diagnosis, and subject to medical follow-up for at least one year after diagnosis. All patients were classified as stage I, II, III, or IV, according to the TNM classifications of the 6th edition of the American Joint Committee on Cancer (AJCC) and the Union for International Cancer Control (UICC) [25].

### Experts’ panel consensus

The technique based on the nominal group or expert panel consensus [26] was used to define the medical and clinic characteristics of a patient diagnosed with NSCLC, which are relevant for cost estimations. For that purpose, a diagnostic-therapeutic guide was defined. This guide combined the hospital patient pathways and the infrastructure, human resources, technology, and services provided at the INCan.

To do so, the following seven events were defined:

1. Outpatient care
2. Operating room care (surgery)
3. Regular (non-critical care) hospitalization
4. Intensive Care Unit (ICU) hospitalization
5. Chemotherapy administration
6. Radiotherapy treatment
7. Palliative care

It is noteworthy that although there is a protocol for smoking cessation in the first and second levels of health-care including brief advice and psychotherapeutic support; there is not a specialized and integrated protocol for giving up smoking cessation of patients with cancer.

Similarly, for each clinical stage, the human and material resources required were specified in terms of quantity and frequency. The medical and nursing procedures for the medical care of each patient were further defined. The panel of experts established the units of measure, the quantity used, and the duration of the prescribed medication.

### Cost estimates

The finance department at the INCan provided cost information regarding the different medical services, materials, medications, and human resources, as well as the current fixed costs of the institution. The cost assessment was conducted from the perspective of the healthcare service provider using the cost of illness (COI) methodology. To determine the costs, a micro-costing model was developed, and the total costs of the disease were incorporated from the unit costs of the services provided using the bottom-up methodology [27,28]. Cost estimates in Mexican pesos were adjusted by inflation and converted into US Dollars using the 2013 FIX exchange rate for foreign transactions (1 USD = 13.06 Mexican pesos).

### Medical event cost estimates

The unit cost estimates determining the total cost of the disease were calculated using an accounting model [29,30]. The accounting model included the annual salary of the human resources, which included social benefits. The capital costs (equipment and installation) were depreciated and prorated according to the equivalent annual cost (EAC) methodology [27,31]. The cost estimates of the medications and required materials were conducted based on the INCan’s consolidated. The unit of measure was hour/activity (hour/consultation, hour/surgery, hour/regular hospitalization, hour/ICU hospitalization, hour/chemotherapy, hour/radiotherapy, and hour/palliative care).

The following equation was applied to estimate the cost of medical events:

$$ ME{C}_{esptnr}={\displaystyle \sum_{r=1}^{n_2}R{U}_{esptnr}\ast U{C}_{esptnr}} $$

Where:

$$ ME{C}_{esptnr}=\mathrm{Medical}\kern0.5em \mathrm{event}\ \mathrm{cost}\ \mathrm{per}\ \mathrm{patient},\ \mathrm{according}\ \mathrm{t}\mathrm{o}\ \mathrm{t}\mathrm{he}\ \mathrm{t}\mathrm{ype}\ \mathrm{o}\mathrm{f}\ \mathrm{disease} $$

$$ R{U}_{esptnr}=\mathrm{Resource}\ \mathrm{utilization}\ \mathrm{f}\mathrm{o}\mathrm{r}\ \mathrm{t}\mathrm{he}\ \mathrm{medical}\ \mathrm{care}\ \mathrm{o}\mathrm{f}\ \mathrm{t}\mathrm{he}\ \mathrm{event},\ \mathrm{according}\ \mathrm{t}\mathrm{o}\ \mathrm{t}\mathrm{he}\ \mathrm{t}\mathrm{ype}\ \mathrm{o}\mathrm{f}\ \mathrm{disease} $$

$$ U{C}_{esptnr} = \mathrm{Unit}\ \mathrm{price}\ \mathrm{o}\mathrm{r}\ \mathrm{cost}\ \mathrm{per}\ \mathrm{r}\mathrm{esource}\ \mathrm{unit}\ \mathrm{used}\ \mathrm{f}\mathrm{o}\mathrm{r}\ \mathrm{patient}\ \mathrm{care},\ \mathrm{according}\ \mathrm{t}\mathrm{o}\ \mathrm{t}\mathrm{he}\ \mathrm{t}\mathrm{ype}\ \mathrm{o}\mathrm{f}\ \mathrm{disease} $$

- *d* = Disease
  - =Disease severity: Stage I, II, III, and IV

*p* = Number of patients

t = Type of medical event: outpatient, operating room, regular hospitalization, ICU hospitalization, chemotherapy, radiotherapy, or palliative care

- *n* = Number of medical events {*Number of events*/*l* = *i*} {1, 2, … *n*_2_}
  - = Resources used during medical care

### Average cost estimate per case

After estimating the costs of the medical event, the average annual cost per patient by NSCLC disease stage was calculated. The average cost per patient according to NSCLC stage was calculated using the following equation:

$$ MEA{C}_{es}=\frac{{\displaystyle \sum_{p=1}^{n_1}{\displaystyle \sum_{n=1}^{n_2} ME{C}_{esptnr}}}}{n_{est}} $$

Where:

- *MEAC*_*est*_ = Medical event average cost
- *MEC*_*esptnr*_ = Medical event cost for one patient, according to disease severity
  - = Number of patients {1,2…*n*_1_}
  - = Number of medical events {Number of events/*l* = *i*}{1, 2, … *n*_2_}
- *n*_*est*_ = Number of patients undergoing the event, according to disease severity

### Case-specific cost estimate, according to disease severity

Once the unit cost of the medical event was obtained, the average annual cost per case was calculated using the frequency of event occurrence reported by the panel of experts. The average cost per patient according to the clinical stage of the disease was obtained using the following equation:

$$ ACD{S}_{es}\frac{{\displaystyle \sum_{p=1}^{n_1}{\displaystyle \sum_{t=1}^8{\displaystyle \sum_{n=1}^{n_2}MEC{P}_{esptnr}}}}}{n_{es}} $$

Where:

- *ACDS*_*es*_ = Average cost, according to disease severity
- *MECP*_*esptnr*_ = Medical event cost per patient, according to the stage of the disease
  - = Patients {1,2,…*n*_1_}

- ***t*** = Type of medical event: outpatient, operating room, regular hospitalization, ICU hospitalization, chemotherapy, radiotherapy, or palliative care
  - = Number of medical events {Number of events/*l* = *i*} {1, 2, … *n*_2_}
  - = Number of patients per event, according to disease severity

### Total cost of disease at the INCan

The total cost was obtained by multiplying the average annual cost per patient by the number of new NSCLC cases in 2009. These costs were weighted according to the patient distribution by medical event and disease stage. In addition, while the study ran between 2009 and 2010, costs were adjusted for inflation and estimated at USD 2013. The total cost of the disease was calculated using the following equation:

$$ TC{D}_e={\displaystyle \sum_{s=1}^6ACD{S}_{es}\ast {N}_{es}} $$

Where:

*TCD*_*e*_ = Total cost of disease

*ACDS*_*es*_ = Average cost, according to disease severity

*N*_*es*_ = Number of patients per event, according to disease severity

### Cost of disease attributable to smoking

The Population Attributable Fraction (PAF) represents the proportion of NSCLC cases that can be attributed to a causal factor, which in this case is smoking [32-35]. Reynales-Shigematsu et al [36] previously estimated the smoking-attributable fraction (SAF) for NSCLC in the Mexican population ages 35 to 65 that were covered by the Mexican Social Security Institute (IMSS, for its initials in Spanish). The weighted SAF value for NSCLC estimated for Mexico was 0.66 (CI 0.58-0.73). This value was used to estimate the NSCLC costs associated with smoking.

### Total smoking-attributable NSCLC cost at the INCan

Finally, to calculate the cost associated with smoking, the NSCLC SAF was multiplied by the total cost of NSCLC using the following equation:

$$ SD{C}_e=TC{D}_e\ast SA{F}_e $$

Where:

*SDC*_*e*_ = Smoking-related cost of disease

*TCD*_*e*_ = Total cost of disease

*SAF*_*e*_ = Smoking-attributable fraction

## Results

Out of a total of 297 patients diagnosed with NSCLC, 1.2% of the cases were detected at an early stage (I and II); 16% had locally advanced disease (stage III); and 82% presented with metastatic disease (stage IV).

The number of patients undergoing different medical events is described in Table 1.

**Table 1:** **NSCLC Total medical events at INCan in 2009**

**Event**	**No.**
**Outpatient care**	297
**Operating room care (surgery)**	20
**Regular (non-critical care) hospitalization**	192
**Intensive Care Unit (ICU) hospitalization**	10
**Chemotherapy administration**	240
**Radiotherapy treatment**	168
**Palliative care**	240

The average length of stay for hospitalized patients with NSCLC was 22 days. The average daily medical care cost per patient was 3,328 USD (1,580-4,501 USD), out of which 2,196 USD (1,043-2,971 USD) was attributable to smoking. The total cost of hospitalization represented only 2% of the total medical care cost (TMCC) attributable to smoking. Chemotherapy was the most costly treatment for patients with stage III and IV NSCLC, with an average annual medical care cost (AAMCC) of 47,768 USD and 83,868 USD, respectively.

The AAMCC per NSCLC patient was 139,801 USD (13,456-144,555 USD), and the AAMCC per NSCLC patient attributable to smoking was 92,269 USD (8,881-95,406 USD), disregarding the clinical stage of the disease.

The patients with stage III and IV NSCLC were the most costly, reaching an AAMCC of 106,186 USD and 144,555 USD, respectively. However, surgical procedures and chemotherapy treatment were the most costly for patients in stages I and II (Table 2).

**Table 2:** **Annual average cost according to clinical stage and medical event per NSCLC patient at INCan. 2013**

**Lung cancer stages +**							
**Event type**	**I**	**II**	**III**	**IV**	**LC**		
	**Annual cost average**				Weighted annual cost average*	**Minimum**	**Maximum**
Outpatient care	$1,280.75	$2,948.96	$15,129.26	$24,606.10	$23,623.40	$1,280.78	$24,606.10
Regular (non-critical care) hospitalization	$1,580.50	$4,501.53	$2,892.15	$3,359.66	$3,328.02	$1,580.39	$4,501.53
Operating room care (surgery)	$10,595.24	$10,816.84	$21,328.31	-	$11,510.31	$10,595.24	$21,328.31
Intensive Care Unit (ICU) hospitalization	-	$2,723.22	$2,723.22	$2,723.22	$2,723.22		
Chemotherapy administration	-	$14,658.14	$47,768.79	$83,868.53	$81,240.02	$14,658.14	$83,868.53
Radiotherapy treatment	-		$9,245.79	$17,522.05	$16,902.06	$9,245.79	$17,522.05
Palliative care	-		$7,099.35	$12,475.91	$12,192.94	$7,099.35	$12,475.91
Case total	$13,456.39	$35,648.68	$106,186.76	$144,555.47	$139,801.87	$13,456.39	$144,555.47

The cost was estimated in Mexican pesos 2009 and was adjusted by inflation for 2013 using the official INPC and inflation rate established by INEGI http://www.inegi.org.mx/sistemas/indiceprecios/CalculadoraInflacion.aspx.

Currency Dec 2013: USA dollar, 1 dollar = 13.06 Mexican pesos. Exchange according to Bank of Mexico (Banxico) information.

**Figure 1:**
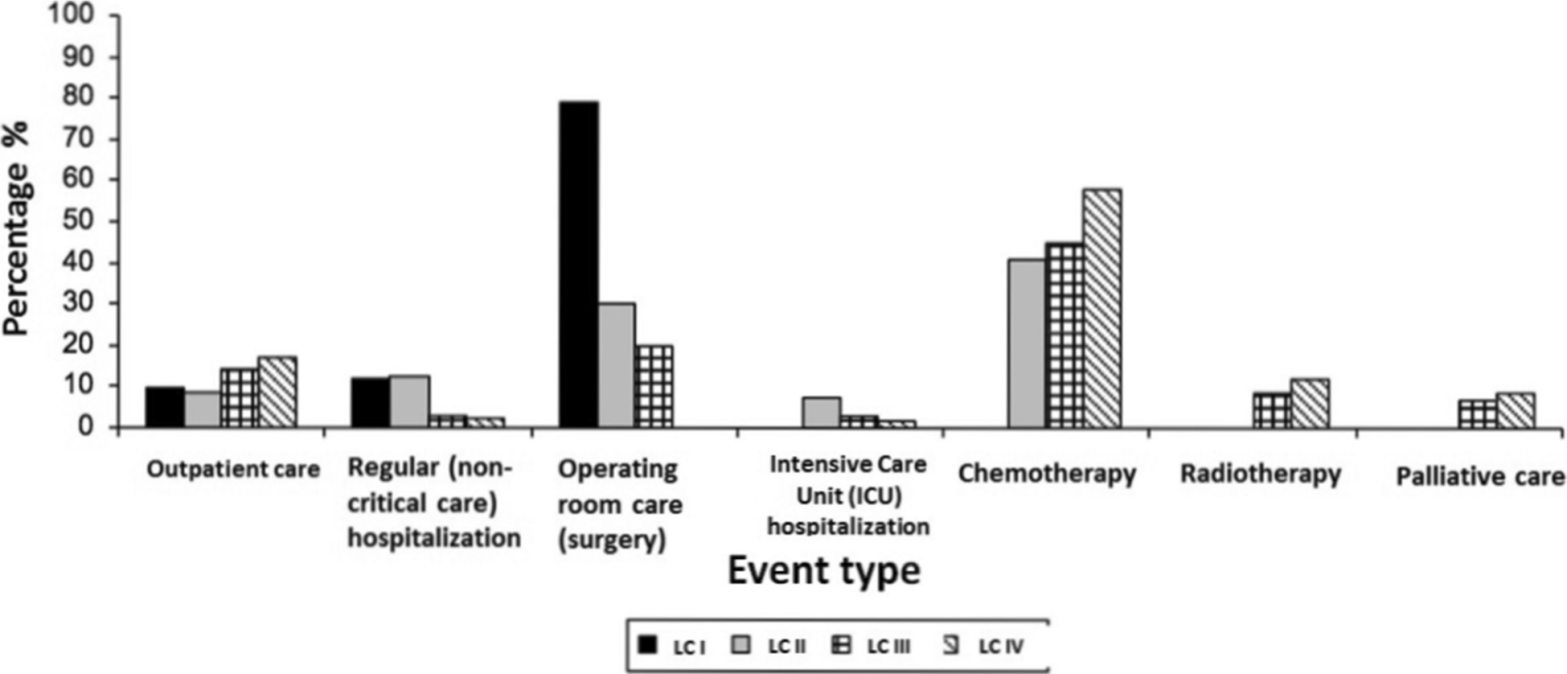
**Proportional costs of different interventions in lung cancer patients by clinical stage.**

For patients with stage I disease, surgical treatment represented 78.7% of the total cost. For stages II and III, surgical treatment and chemotherapy represented 71.5% and 65.1% of the total annual cost, respectively. For stage IV, outpatient consultations and chemotherapy represented 75.0% of the total costs (Figure 1).

The TMCC for patients with a first-time diagnosis of NSCLC was 30,257,245 USD, while the TMCC attributable to smoking was 19,969,781 USD (Table 3).

**Table 3:** **Total costs according to NSCLC clinical stage at INCan. 2013**

**Stage** ^**+**^	**Annual total cost**	**Annual total cost due to tobacco** ^*^
LC I	**$48,617.17**	**$32,087.28**
LC II	**$80,957.16**	**$53,431.72**
LC III	**$985,634.28**	**$650,518.63**
LC IV	**$29,142,036.54**	**$19,233,744.07**
Total	**$30,257,245.15**	**$19,969,781.81**

The cost was estimated in Mexican pesos 2009 and was adjusted by inflation for 2013 using the official INPC and inflation rate established by INEGI http://www.inegi.org.mx/sistemas/indiceprecios/CalculadoraInflacion.aspx.

Currency Dec 2013: USA dollar, 1 dollar = 13.06 Mexican pesos.

Exchange according to Bank of Mexico (Banxico) information.

^+^Structure established by the Lung Cancer experts of the INCan and the CIE-10 2009.

^*^Obtained with tobacco attributable fraction of LC, calculated for national IMSS; 0.66. Reynales et al.

For the same calendar year, stage IV LC due to smoking was the most costly, representing 63.6% of the TMCC.

An interesting fact is that the medical care costs in public hospitals, such as the INCan, are split between the patient and the institution. The division is based on a payment level category assigned to the patient: the patient’s payments increase proportionally with the treatment costs, thus decreasing the institutional costs.

**Figure 2:**
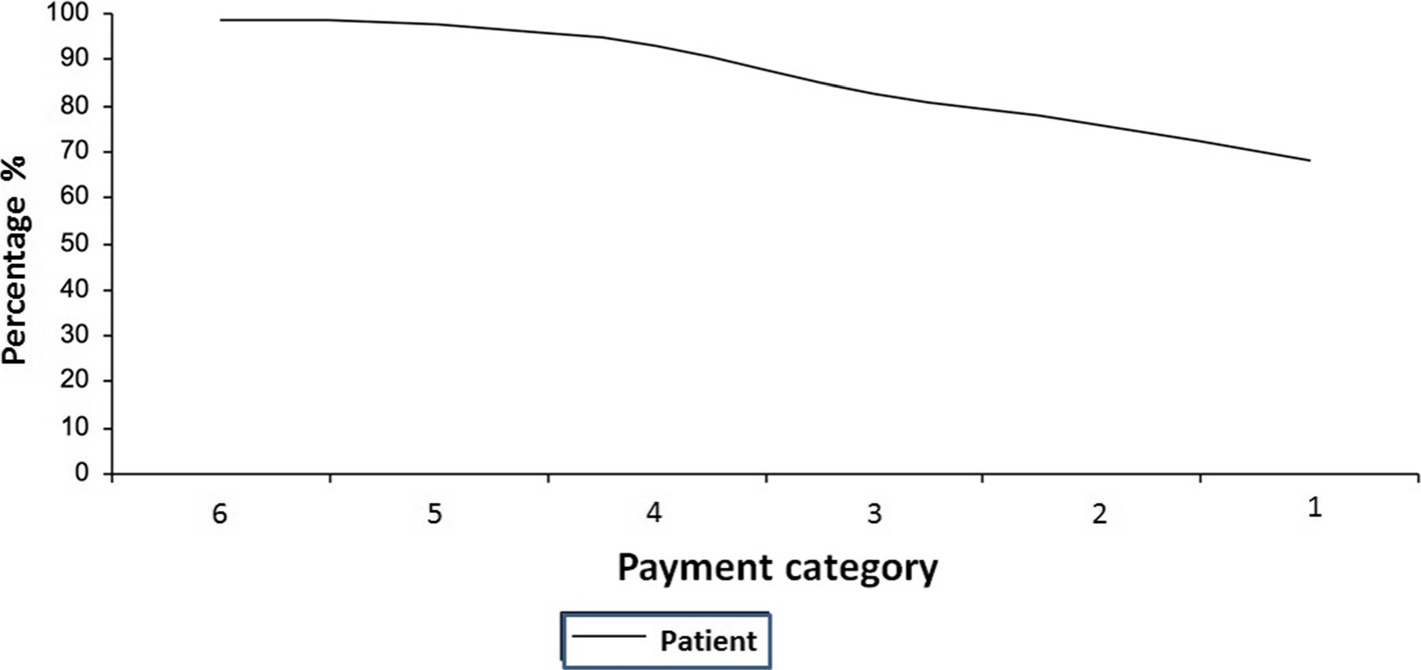
**Proportion of lung cancer patients in each payment category at INCan.**

Despite some patients being classified as level 1, the patient’s cost percentage is approximately 70%, given that medication costs (which account for most of the costs) are not subsidized by the INCan (Figure 2).

## Discussion

This study provides the first detailed cost estimate for the medical care of NSCLC attributable to smoking at an oncology center in Mexico. The results of this study show that the annual costs attributable to smoking are high for patients with newly diagnosed NSCLC, exceeding 18 million USD (235 million Mexican pesos). The medical care costs for recently diagnosed LC patients are mainly due to surgical and chemotherapy treatments.

In Mexico, during 2013, the medical care costs for NSCLC attributable to smoking, as estimated by the Social Security Administration of the Mexican Secretariat of National Defense (SEDENA, for its initials in Spanish), were 2.1 million USD (28.4 million Mexican pesos, calculated based on an exchange rate of 1 USD = 13.06 Mexican pesos, according to the information provided by the Bank of Mexico [Banxico] for 2013). That same year, the Mexican Institute for Social Security and Services for State Workers (ISSSTE, for its initials in Spanish) estimated the costs associated with NSCLC attributable to smoking to be 2.8 million USD (36.7 million Mexican pesos) [37].

The differences in the total treatment costs for NSCLC in third-level hospitals in Mexico (IMSS, ISSSTE, and SEDENA), which were calculated using the same cost estimate methodology, are mainly due to changes in unit costs and the frequency of use of the different resources. Direct cost accounting varies with the level of human resource specialization, the implementation of diagnostic and treatment guidelines, and the unit costs of medication and equipment (e.g., unit costs for stents) at each institution. Indirect cost accounting varies with the available hospital infrastructure (e.g., number of beds for hospitalization purposes, operating rooms, and intensive care units) and the high-cost technology used for diagnostic and treatment procedures. In addition to the aforementioned data, it must be taken into consideration that the analysis of every epidemic must include the disease incidence and prevalence rates, as well as its etiology and its impact on population health. Tobacco addiction can be considered the worst-ever epidemic for humanity. Thus, the most appropriate way to approach it is not only by recognizing its health consequences but also by understanding how the tobacco industry manipulates the most vulnerable groups, altering the image of the disease-causing agent [12,38].

In the last 100 years, lung cancer went from being a medical rarity to the main cause of death from cancer in the world. This increase was achieved by the introduction of smoking into our society during the first half of the 20th century, when the proportion of male smokers was as high as 60% [38,39]. According to the National Addiction Survey of 2011 in Mexico, the prevalence of smokers in the population (ages 12 to 65) was 21.7% [16]. This percentage represents a population of 17.3 million Mexican smokers. In 1989, only 25% of smokers started smoking at age 12, while in 2011, this fraction had increased to 50%. That same year, 25,383 deaths due to smoking were registered, of which 5,615 were due to cancer. These results suggest that smoking is the main etiological factor for the top 10 causes of morbidity and mortality in Mexico [16].

Despite the existing sub-registry, the Mexican National Institute of Statistics and Geography (INEGI, for its initials in Spanish) reported that in 2008, there were more than 9,000 LC-related deaths in our country. This number represents the second most frequent cause of cancer-related death in males and the third most frequent one in women, after prostate and breast/uterine cancer, respectively [40]. In contrast to developed countries, such as the United States (U.S.) or Europe, where 85% of LC is associated with smoking, only 66% of LC cases in our country were found to be due to smoking. While there are other risk factors, such as wood smoke exposure [18,41], second-hand smoke [18,42], and tuberculosis [43], the role of tobacco as a disease-causing agent cannot be overlooked.

If we compare NSCLC to breast and colorectal cancer, where advances in treatment have doubled the patient survival rates [9,10,44,45], the impact of treatment on the survival of patients with NSCLC is limited. In the 1960s, the 5-year survival rate was 12%, while it is currently 16% [42]. Furthermore, smoking is not only associated with LC development but is also strongly linked to genetic alterations within the lung tumors themselves, such as K-Ras mutations [46], which turn them into more aggressive cancers with poor response to treatment [47].

Even if the present study did not take them into account, in recent years, the use of targeted therapies has been shown to improve the survival of NSCLC patients with specific mutations that make them ideal candidates for said treatments [46,48-52]. However, these medications are extremely costly and, generally, poorly accessible to the Mexican population. This problem is particularly serious in a developing country, such as ours, where the cost of medications is similar to their cost in developed countries. For example, the cost of erlotinib 150 mg (an epidermal growth factor receptor [EGFR] inhibitor) is 3,100 USD in the U.S. and close to 3,000 USD in Mexico. However, the minimum daily wage for 2013 in the U.S. is approximately 9 USD/hour [53], while in our country, it is 0.57 USD/hour [54]. This difference deepens the existing inequality for the optimal management of LC patients associated with the cost of these types of therapy.

Worldwide, tobacco control policies have evolved as evidence of the harmful health effects of smoking has arisen. The objectives of these policies are to keep the non-smoking population from consuming tobacco while helping active smokers quit this habit. These policies include information and education about the risks of smoking, smoking bans in public spaces, blocking smoking advertisements on television, adding warning labels to cigarette packs, and raising taxes on tobacco producers. These public policy measures have decreased smoking in several developed countries [38,55]. Unfortunately, the opposite has happened in developing countries, where the number of smokers has increased, especially among women and teenagers. This increase is mainly due to the tactics employed by the tobacco industry and the lack of effective public policies [1,8,56-58].

Finally, in order to address the generalizability of the study is is important to mention that, in Mexico, health care is mainly delivered throughout three institutions: the Mexican Institute for Social Security (IMSS), the Institute for Social Security and Services for Civil Servants (ISSSTE) and the Health Department (SSA). The IMSS is responsible for the health care of 40% of the population, whereas the ISSSTE attends 35% and the SSA the remaining 25% of the mexican population. The INCan, belongs to the health department. However, is noteworthy that there are significant differences for NSCLC treatment among those institutions. Also, there are several strengths to this study; it is the first cost estimate study for NSCLC using the COI methodology in a third-level medical service center in Mexico covering the population without social security benefits; employing a more precise methodology for estimating costs, given that it used the information in the medical records of patients. This information allowed a greater precision in the diagnosis and classification of cases, as well as in the actual frequency of use of different medical resources. Our study used financial information to estimate the medical care costs due to smoking at an institutional and national level.

Our results provide a powerful argument that endorses and supports public health policies against smoking. Our cost information is useful and necessary for estimating the national budget and how it will be utilized. The health sector needs to invest in the treatment and prevention of NSCLC over the next decades. Lastly, our results will allow future cost-effectiveness and cost-benefit studies to be conducted, which will allow assessment of the economic impact of tobacco control policies in Mexico over the last 5 years. The medical care costs of NSCLC attributable to smoking at the INCan are high. These increased costs should prompt the authorities to implement strategies and prevention programs following the better practices recommended by the WHO-FCTC. The prevention and control of all modifiable risk factors related to NSCLC (wood smoke, alcohol abuse, and smoking) will not only benefit morbidity and mortality rates but will also represent an important reduction in healthcare costs at a national level.

This study illustrates the high cost of medical care for a disease that is largely attributable to smoking. A malignant tumor can be prevented in more than 80% of cases. The medical care costs reported in this study are catastrophic for any patient without health insurance. It should be noted that this analysis took only tangible costs into account. Indirect costs due to premature death, early retirement, and loss of productivity, among other factors, were not further studied [36,37,59]. In Mexico, it is imperative to implement better and more aggressive tobacco control policies to reduce the number of deaths that are attributable to smoking.

## Conclusion

The medical care costs incurred by patients with lung cancer and a smoking history represent a heavy burden for both the INCan and the Mexican health care system. Efforts to enforce the provisions established in the Framework Convention of Tobacco Control of the World Health Organization should be pursued in order to significantly reduce these costs.
